# Editorial: Identifying Individuals at Clinical High Risk of Psychosis in Different Cultures and Countries

**DOI:** 10.3389/fpsyt.2020.00159

**Published:** 2020-02-28

**Authors:** TianHong Zhang, JiJun Wang, Kristen A. Woodberry

**Affiliations:** ^1^Shanghai Mental Health Center, Shanghai Jiaotong University School of Medicine, Shanghai Key Laboratory of Psychotic Disorders, Shanghai, China; ^2^Bio-X Institutes, Key Laboratory for the Genetics of Developmental and Neuropsychiatric Disorders (Ministry of Education), Shanghai, China; ^3^Brain Science and Technology Research Center, Shanghai Jiao Tong University, Shanghai, China; ^4^Center for Psychiatric Research, Maine Medical Center Research Institute, Portland, ME, United States

**Keywords:** ultra high risk (UHR), transition, identification, prevention, prodromal psychosis

Identifying individuals at clinical high risk (CHR) for psychosis leverages a critical window of opportunity for prevention and early intervention. The characterization of effective detection and therapeutic strategies for this population represents one of the most unmet needs of contemporary psychiatry. The purpose of this Research Topic is to reflect on the similarities and differences in clinical, cognitive, biological, cultural and social aspects of CHR samples from different cultures and countries. This topic issue presents 3 perspectives, 1 study protocol, 4 reviews, and 4 original research articles which span the field of CHR research in different countries and offer insightful directions for future study and comprehensive practical suggestions in improving the efficacy in early intervention.

## Perspectives

All three perspective pieces speak to the degree to which current CHR methods are identifying and serving the actual target population. Schiffman et al., argue that the current “one-size-fits-all” approach of CHR identification does not fully reflect individual differences, particularly in context, ethnicity, race, culture, and development. They propose practical strategies for improving the accuracy of CHR identifications within and across different cultural settings. A culturally specific example, by Parabiaghi et al., describes the implementation of early detection and treatment of severe mental illness in youth across multiple regions of Italy. Promoting local community coalitions and an emphasis on accessibility, their broad preventive approach identified a group of 15–24 year olds enriched for CHR status. Finally, Kennedy et al., discuss what is needed from a public health perspective to extend systematic screening for early psychotic symptoms to general practice clinics.

## Protocol

Mahmood et al., describe a specific protocol to test a novel intervention in an underserved Latino CHR population in two different languages (Spanish and English) and countries (the United States and Mexico). This efficacy pilot is comparing Compensatory Cognitive Training (CCT) with recreational therapy (RT) to target cognitive and functional outcomes. Trials that extend the inclusiveness of the population served and real-life outcomes measured have important implications for the relevance of CHR efforts to public health.

## Reviews

Improving the detection of CHR-P individuals is the topic of three reviews. Oliver et al. review the limitations of current structured interviews for identifying CHR and propose to address them with a Psychosis Polyrisk Score (PPS) prototype based on non-genetic risk factors, including social context. In a conceptual but non-systematic review furthering attention to sampling biases, Fusar-Poli et al., illustrate risk detection models targeting three different populations: secondary mental health care, primary care, and the community (general population). From their review of the evidence, the authors argue for the international advancement of CHR detection through complementary approaches. Transdiagnostic individualized risk calculators must be tested and implemented in primary and secondary care and digital and/or sequential screening in community samples. The final review on this topic extends the literature covered to include the prediction of outcomes in individuals identified using established structured interviews of CHR. Based on a meta-analysis of the largest sample of individualized data (*n* = 1,676), their model achieved only moderate prognostic value. They argue that the high level of heterogeneity in samples worldwide limits the clinical value of any one predictive model.

Finally, since the 0 to 25 years is a vulnerable developmental period during which children and young people experience many psychosocial and neurobiological changes, Fusar-Poli, on behalf of the Healthy London Partnership, reviews the evidence for established integrated and youth-friendly mental health services. In spite of the lack of robust controlled trials on their impact, early intervention for psychosis services may provide a paradigm to lead further reform.

## Original Research

Two original research studies investigate clinical and behavioral characteristics of early psychosis in culturally diverse samples. A study from the Korean Early Psychosis Cohort by Won et al., explores the characteristics and patterns of emotional recognition deficits in 495 patients with early psychosis. Their results show the correlation between symptom severity and the extent of emotional recognition deficits for different emotions. Examining the clinical characterization of schizotypy dimensions in a largely adolescent student sample (*n* = 1,506) from northern Spain, Fonseca-Pedrero et al., estimates a multidimensional psychosis liability network, a dynamic and complex system of risk and protective factors.

Two other studies target the biological correlates of CHR syndromes and symptoms. Liu et al., investigates the resting-state functional connectivity of the alpha rhythm measured by electroencephalography (EEG) to test a hypothesis of abnormal functional connectivity. Both early psychosis groups (first-episode schizophrenia and CHR) show an increased degree of connectivity compared with healthy controls, especially in the left occipital lobe area which is higher in the CHR group than in the first-episode schizophrenia group. Bonoldi et al., examine the relationship between basic self-disturbances and alterations in cortical midline structure volume measured by magnetic resonance imaging (MRI). They find that the higher level of basic self-disturbances in CHR individuals appear to be related to reductions in anterior cingulate volume.

Taken together, the high-quality contributions gathered in this Research Topic highlight both the promise and limitations of extant research for identifying, understanding, and helping CHR individuals across different cultures and countries. Several important and exciting efforts have been completed or are in progress, but much work still remains to be done. These articles provide international research collaborators with the key insights to further improve the tools and methods for identifying CHR individuals, and for developing effective interventions as well.

## Author Contributions

TZ, JW, and KW have contributed intellectual content and have contributed to the actual writing of the editorial.

### Conflict of Interest

The authors declare that the research was conducted in the absence of any commercial or financial relationships that could be construed as a potential conflict of interest.

